# Tau pathogenesis is promoted by Aβ1-42 but not Aβ1-40

**DOI:** 10.1186/1750-1326-9-52

**Published:** 2014-11-23

**Authors:** Xiaoyan Hu, Xiaoling Li, Mingrui Zhao, Andrew Gottesdiener, Wenjie Luo, Steven Paul

**Affiliations:** Appel Alzheimer’s Disease Research Institute, Weill Cornell Medical College, 413 East 69th Street, 10th Floor, BB 1051, mailbox #240, New York, NY 10065 USA; Strang Laboratory of Apoptosis & Cancer Biology, Rockefeller University, 413 East 69th Street, 10th Floor, BB 1051, mailbox #240, New York, NY 10065 USA; Department of Neurological Surgery, Weill Cornell Medical College, 413 East 69th Street, 10th Floor, BB 1051, mailbox #240, New York, NY 10065 USA

**Keywords:** Aβ1–42, Aβ1–40, tau, Alzheimer’s disease, Aggregation, Phosphorylation, Cleavage

## Abstract

**Background:**

The relationship between the pathogenic amyloid β-peptide species Aβ1–42 and tau pathology has been well studied and suggests that Aβ1–42 can accelerate tau pathology in vitro and in vivo. The manners if any in which Aβ1–40 interacts with tau remains poorly understood. In order to answer this question, we used cell-based system, transgenic fly and transgenic mice as models to study the interaction between Aβ1–42 and Aβ1–40.

**Results:**

In our established cellular model, live cell imaging (using confocal microscopy) combined with biochemical data showed that exposure to Aβ1–42 induced cleavage, phosphorylation and aggregation of wild-type/full length tau while exposure to Aβ1–40 didn’t. Functional studies with Aβ1–40 were carried out in tau-GFP transgenic flies and showed that Aβ1–42, as previously reported, disrupted cytoskeletal structure while Aβ1–40 had no effect at same dose. To further explore how Aβ1–40 affects tau pathology in vivo, P301S mice (tau transgenic mice) were injected intracerebrally with either Aβ1–42 or Aβ1–40. We found that treatment with Aβ1–42 induced tau phosphorylation, cleavage and aggregation of tau in P301S mice. By contrast, Aβ1–40 injection didn’t alter total tau, phospho-tau (recognized by PHF-1) or cleavage of tau, but interestingly, phosphorylation at Ser^262^ was shown to be significantly decreased after direct inject of Aβ1–40 into the entorhinal cortex of P301S mice.

**Conclusions:**

These results demonstrate that Aβ1–40 plays different role in tau pathogenesis compared to Aβ1–42. Aβ1–40 may have a protective role in tau pathogenesis by reducing phosphorylation at Ser^262^, which has been shown to be neurotoxic.

## Background

Two major hallmarks of Alzheimer’s disease (AD) are the accumulation of the amyloid-β peptides (Aβ) into extracellular plaques and the formation of intracellular neurofibrillary tangles (NFTs) composed mainly of the protein tau. Aβ exists as two main species, Aβ1-42 and Aβ1-40, and the unique relationship between each of these peptides on tau pathology has not been adequately addressed. It is generally believed that Aβ accrual in brain is an early event in the pathogenesis of AD, preceding significant tau pathology. Intracerebral administration of synthetic Aβ1-42 fibrils into P301L mice (mutant human tau transgenic mice) induced tau hyperphosphorylation and local neurofibrillary tangles [1, 2]. Natural Aβ oligomers, specifically dimers, isolated from a human AD brain, were sufficient to induce tau hyperphosphorylation at AD-relevant epitopes as well as microtubule disruption and neuritic degeneration [3]. These experiments were done either with Aβ1-42 or a mixture of Aβ1-42 and Aβ1-40; however, increasing evidence suggests that Aβ1-40 and Aβ1-42 differentially contribute to the disease process [4]. BRI-Aβ40 mice, a model that exclusively expresses high levels of Aβ1-40, do not develop amyloid pathology in the form of diffuse or neuritic Aβ plaque. BRI-Aβ42 mice, on the other hand, a model that exclusively expresses Aβ1-42, and at levels 10-fold lower than BRI-Aβ40 mice express Aβ1-40, developed amyloid deposits in the cerebellum as early as 3 months of age [4]. By crossing BRI-Aβ40 or BRI-Aβ42 mice with Tg2576 (APPswe, K670N + M671L) mice, it has been shown that Aβ1-42 and Aβ1-40 have opposing effects on amyloid deposition; Aβ42 promotes amyloid deposition, while Aβ40 inhibits it [5]. In addition, inhibition of angiotensin-converting enzyme (ACE), which converts Aβ42 to Aβ40, enhanced brain amyloid deposition in Tg2576 mice [6]. Emerging evidence therefore indicates a protective role for Aβ1-40 in AD pathogenesis. One such theory suggests that Aβ1-40 may act to stabilize Aβ1-42 monomers by competing for binding sites on pre-existing Aβ1-42 aggregates [7] and thereby inhibiting further aggregation of Aβ1-42 [8].

Aβ1-42 has also been shown to increase the phosphorylation and aggregation of tau both in vivo [1] and in vitro [3, 9]; however, the interaction between Aβ1-40 and tau has not, to our knowledge, been explored. Increasing experimental evidence on differential (in some cases opposite) roles that Aβ1-40 and Aβ1-42 may play in amyloid deposition raises the question of whether Aβ1-40 alters tau phosphorylation and/or aggregation in the same manner as Aβ1-42. Here, we address this issue by using: 1) a cell-based model to study the effects of Aβ1-42 and Aβ1-40 on tau aggregation using live cell imaging; 2) a transgenic Drosophila model to examine the direct impact on cytoskeleton structure caused by Aβ1-42 and Aβ1-40; 3) a tau transgenic mouse model to compare the effects of Aβ1-42 and Aβ1-40 on tau pathogenesis. Here we show that Aβ1-42 induces tau phosphorylation, aggregation and cleavage whereas Aβ1-40 does not.

## Results

### Full-length tau aggregates quickly in SHSY5Y and C17.2 cells, but not in N2a cells

**Figure 1:**
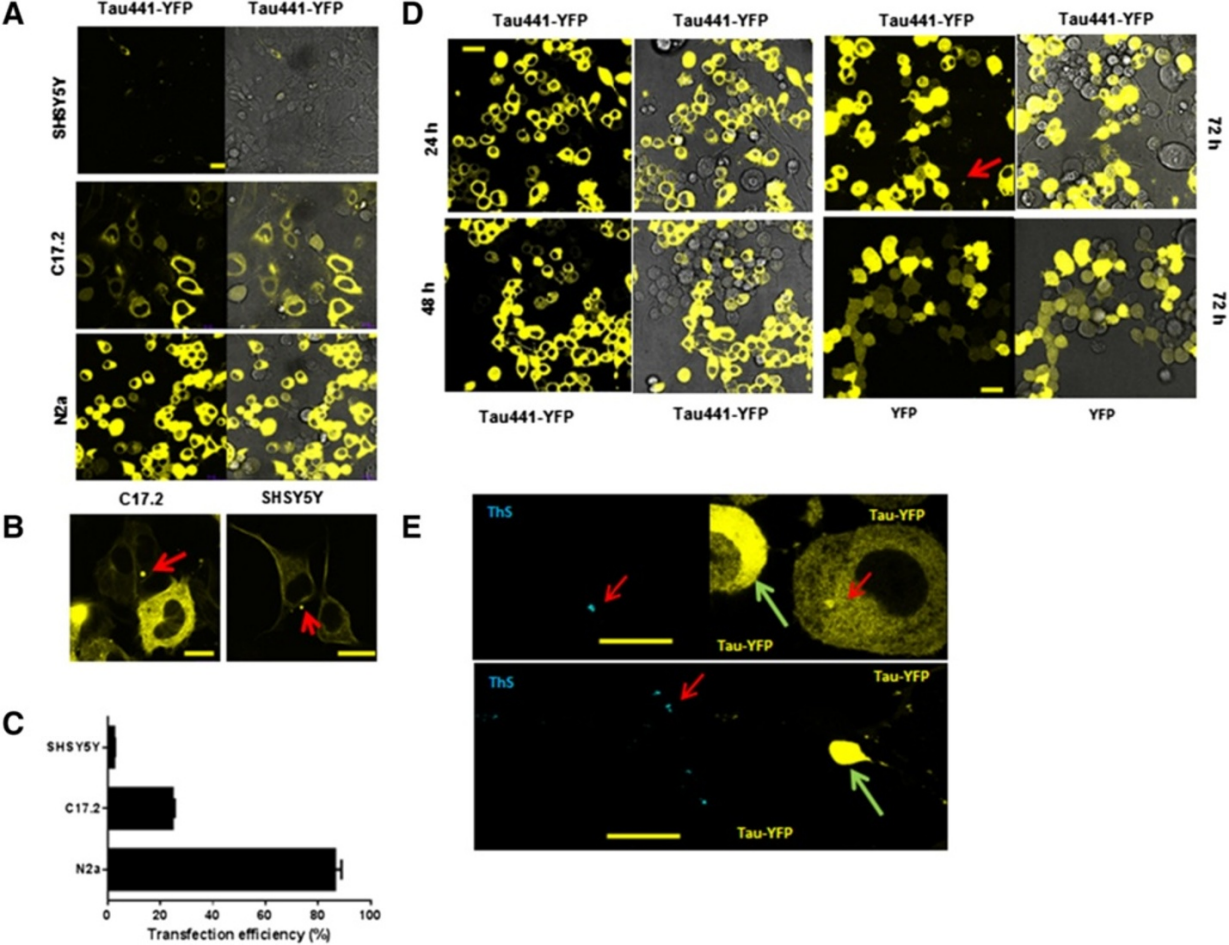
**Expression and aggregation of Tau441-YFP in three different cell types. A)** SHSY5Y, C17.2 and N2a cells were transfected with Tau441-YFP and live cell images were taken at 24 h with a confocal microscope. Darkfield images showed better fluorescence signal and phase contrast provided images for the whole cell. **B)** Aggregates formed in SHSY5Y and C17.2 cells (red arrow). **C)** Quantification of transfection efficiency was done with Image J. **D)** Time course of Tau441-YFP expression and aggregation in N2a cells. Aggregates appeared at 72 h after transfection while no aggregates formed in YFP transfected cells, which suggested that those aggregates were tau specific. **E)** 72 h after transfection, cells were fixed with 4% PFA and stained with thioflavin-S (ThS). Only a small population of cells showed ThS-positive aggregates (red arrow). Cells (green arrow) with diffusely distributed tau were ThS negative even though a high level of Tau441-YFP was expressed in those cells. This further confirmed that the ThS signal was not a false signal due to the leakage of YFP. Magnification: 63x. Scale bar: 10 μm.

To explore the capability of wild-type full-length tau (2N4R, Tau441) to aggregate in living cells, SHSY5Y, C17.2 and N2a cells were plated on an ibidi u-Slide 8 well chamber and transiently transfected with Tau441-YFP (2N4R—longest form of human tau) using lipofectamine 2000. Twenty four hours later, live cells images were taken using a confocal microscope (see Methods). Darkfield and phase contrast views of cells were shown side to side. Darkfield images showed better fluorescence signal (especially for intracellular aggregates) and phase contrast provided images for the whole cell. As shown in Figure 1A, after 24 h we observed the aggregation of full length tau in the vesicles of C17.2 cells and SHSY5Y cells (Red arrow in Figure 1B) but not in N2a cells, suggesting cellular specificity for the aggregation of tau. This is the first study to show that wild-type full-length tau can form aggregates without the addition of exogenous recombinant tau fibrils. By quantifying the fluorescent intensity of YFP linked to Tau441, we calculated the transfection efficiency in N2a cells to be approximately 85% compared to 35% for C17.2 and 5% for SHSY5Y cells.To examine the aggregation of Tau441 in N2a cells over time, live cell images were taken at 24 h, 48 h and 72 h after transfection. Tau aggregates were seen in N2a cells 72 h after transfection (Figure 1D), but not in YFP expressing cells, indicating that the aggregates seen in tau-YFP transfected N2a cells were not YFP-dependent, but specific for tau. Thioflavin-S (ThS) was used to determine if tau aggregates were in β-pleated sheets structure as seen in tau transgenic mice and human AD brain sections. Seventy two hours after transfection with Tau441-YFP, N2a cells were fixed and stained with ThS. As shown in Figure 1E, ThS staining completely overlapped with the large tau aggregates highlighted with YFP, but not with diffusible tau in cytoplasm.

### Aβ1-42 increases the level of insoluble tau while Aβ1-40 has no effect

**Figure 2:**
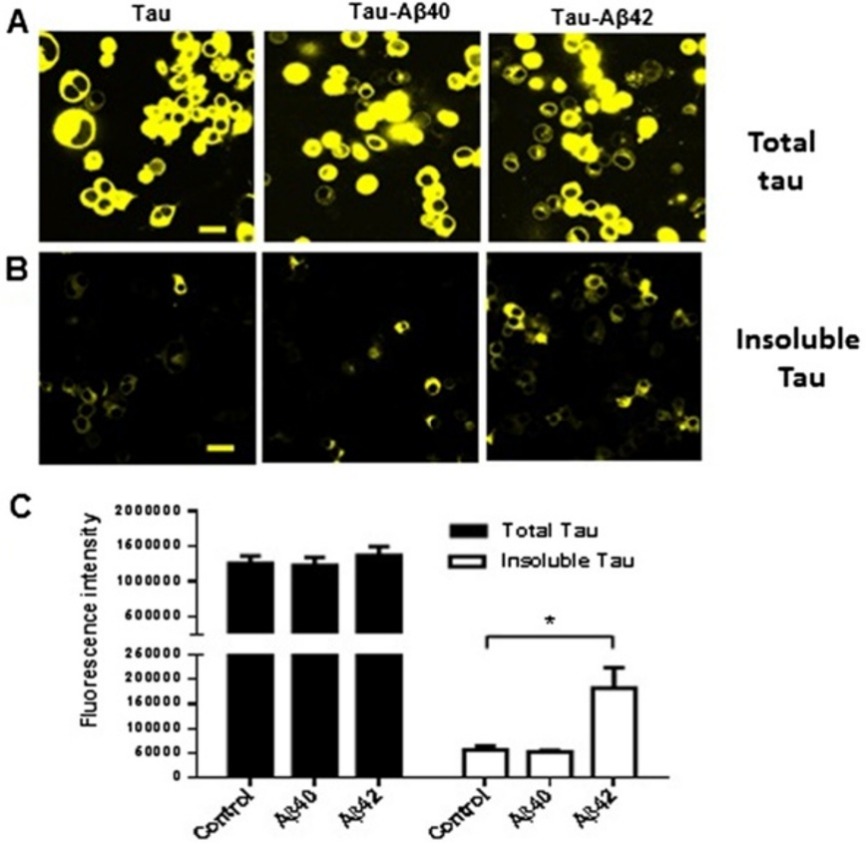
**Aβ** **1-42 induces intracellular full length tau (Tau441-YFP) aggregation in N2a cells. A)** N2a cells were transfected with Tau441-YFP and treated with 200nM Aβ1-40 or Aβ1-42. 24 h later, cells were imaged directly under a confocal microscope. There was no difference in the expression of tau between the groups. **B)** Cells were extracted with 1% Triton during fixation, which left only insoluble tau in the cells. As shown in Figure B, Aβ1-42 treatment increased the level of insoluble full-length tau, but Aβ1-40 did not. **C)** Quantification of fluorescence intensity was done with Image J. Magnification: 63x, scale bar: 10 μm. Three individual experiments were done for each condition. *p <0.05.

In vivo, Aβ1-42 is more prone to forming amyloid plaque than Aβ1-40. In fact, Aβ1-40 has been shown to have a protective effect by reducing the fibrilization of Aβ1-42 [5]. Using the cell-based model we established above, we first determined the respective influence of Aβ1-40 and Aβ1-42 on tau insolubility. N2a cells were transfected with Tau441-YFP in the presence of 200 nM soluble Aβ1-40 or Aβ1-42. Twenty four hours after transfection, live cell images were taken using confocal microscopy and there was no difference in the total fluorescence intensity between the groups (Figure 2A). To assess the effects of Aβ on tau insolubility, cells were fixed with 4% PFA containing 1% Triton-X100 for 15 min to remove soluble tau from the cells. Images shown in Figure 2B, where the fluorescent signal represents insoluble tau, indicate that there was much more insoluble full-length tau in the presence of Aβ1-42 when compared to Aβ1-40 and non-treated cells. Quantification of the fluorescence intensity in these images was done using Image J (Figure 2C). Cytotoxicity was found in Aβ1-42 treated N2a cells at 72 hr but not in Aβ1-40 treated cells (data not shown).

### Aβ1-42 induces cleavage, phosphorylation and aggregation of tau while Aβ1-40 does not

**Figure 3:**
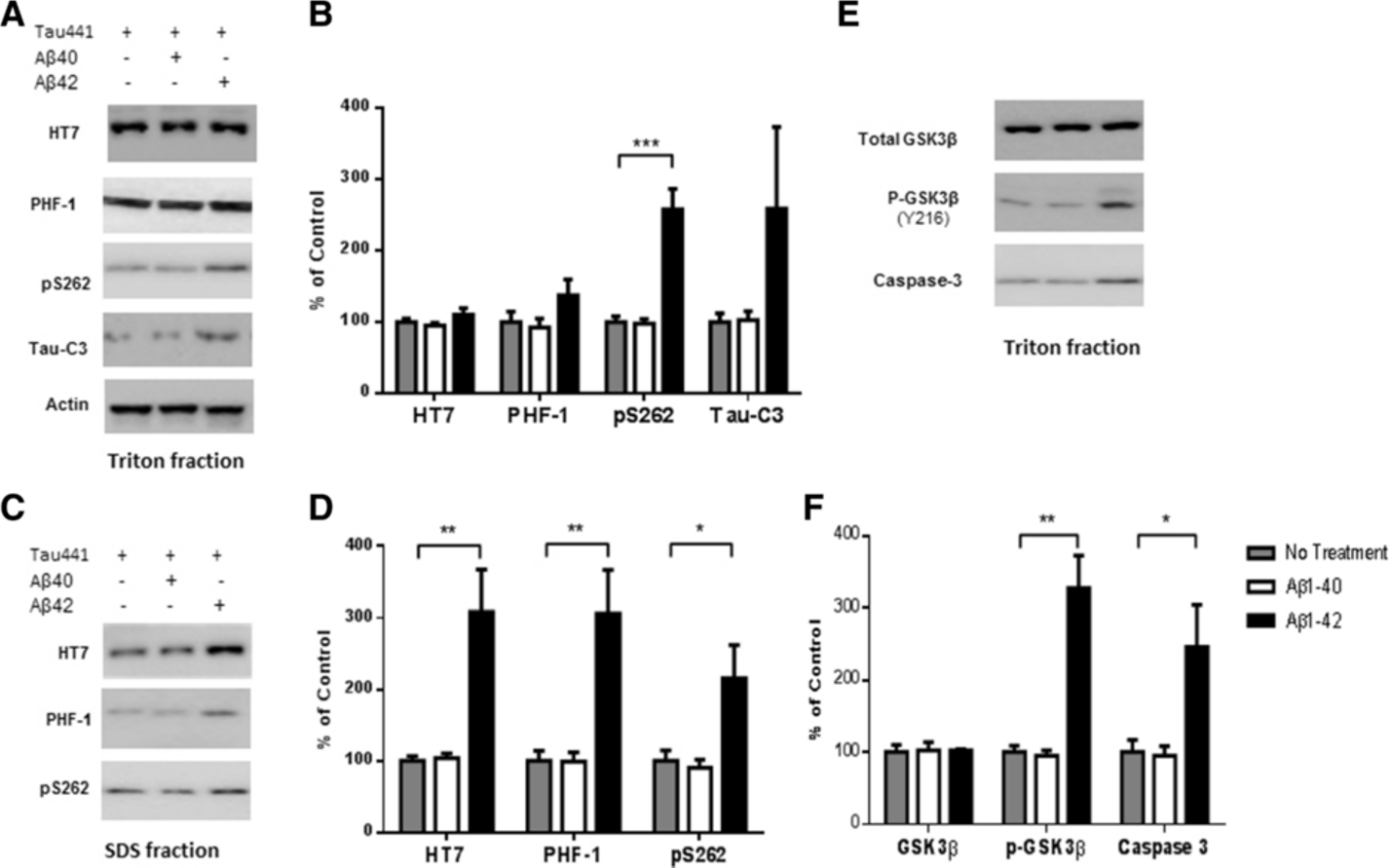
**A** **β1-42 promotes phosphorylation, cleavage and aggregation of tau by increasing GSK3β** **activity and by activating pro-caspase3.** Sequential extraction was performed on Tau441-YFP-transfected cells treated with transfection reagent alone (control), 200 nM Aβ1-40 or Aβ1-42 for 24 h. Triton fraction was obtained by extracting intracellular tau with 1% Triton. Triton-insoluble fraction was then solubilized with 1% SDS to get SDS fraction. **A)** Triton fraction was electrophoresed and immunoblotted for total tau (HT7), p-tau(Ser^396/404^) (PHF-1), p-tau(Thr^262^) (pS262), Tau421(tau-C3) and β-actin. β-Actin was measured from the same blots to ensure that equal protein was present in every lane. Aβ1-42 significantly induced p-tau at Thr^231^ and increased levels of Tau421 were observed in Aβ1-42 treated cells, while no change occurred in Aβ1-40 treated cells when compared to cells without treatment. **B)** Quantification of western blots in **(A)**. **C)** Representative images of western blots for SDS fraction with HT7, PHF-1 and pS262. All 3 forms of tau increased in Aβ1-42 treated cells. No change in tau level was found in Aβ1-40 treated cells. There was no detectable signal for tau-C3 in SDS fraction. **D)** Quantification of western blots in **(C)**. **E)** Triton fraction was also blotted with anti-GSK3β antibody, anti-p-GSK3β (Y216) and anti-caspase-3 antibody. Aβ1-42 treatment resulted in an increase in phosphorylation of GSK3β, which represents higher GSK3β activity. More active caspase-3 was detected in Aβ1-42 treated cells. **F)** Quantification of western blots in **(E)**. Histographs show mean +/− standard error, n =3. *p <0.05; ** p <0.01; ***p <0.005.

To understand the underlying mechanisms that contribute to the unique roles of Aβ1-42 and Aβ1-40 on tau pathology, N2a stable cell line that overexpresses Tau441-YFP was established. Twenty four hours after treatment with either Aβ1-42 or Aβ1-40, the cells were homogenized on ice in 1% Triton-PBS buffer, followed by 1% SDS-PBS buffer. Western blots analysis of Triton and SDS extract using anti-tau antibodies showed that Aβ1-42 treatment resulted in increased phosphorylation at Ser^262^, as recognized by the pS262 antibody, in both Triton and SDS soluble fractions as compared with N2a cells that did not receive Aβ treatment (Figure 3A and C). A slight decrease in the pS262 signal was observed in the SDS fraction from Aβ1-40 treated cells. Phosphorylation at Ser^262^ strongly reduces binding of tau to microtubules and has been shown to be a key phosphorylation site implicated in the loss of function of tau in cytoskeleton stabilization [10]. Ser^396^/Ser^404^ is important phosphorylation site that is recognized by the PHF-1 antibody. In Aβ1-42 treated cells we also observed a 3-fold increase in total tau and phosphorylation at the PHF-1 epitope (Ser^396^/Ser^404^) in the SDS fraction, but we observed no change in Aβ1-40 treated cells (Figure 3B and D). We also observed an increase in Tau421 (cleavage of tau at Asp^421^) in the Triton fraction after Aβ1-42 treatment as visualized with the tau-C3 antibody (Figure 3A and B). No tau-C3 signal was detected in the SDS fraction. Our data are consistent with previous studies on the accelerating effect of Aβ1-42 on tau pathology. To our knowledge, this is the first study to show that Aβ1-40 has no effect on tau phosphorylation, cleavage and aggregation.

### Activation of GSK3β and caspase-3 by Aβ1-42 but not by Aβ1-40

GSK3β phosphorylates tau at many sites including Ser^262^, Ser^396^ and Ser^404^. To understand the underlying mechanism responsible for the differences in tau phosphorylation induced by Aβ1-42 and Aβ1-40 treatments, total GSK3β and phosphorylated-GSK3β (p-GSK3β) levels were examined by western blot. Phosphorylation of GSK3β at Tyr^216^ increases the activity of GSK3β, which leads to more tau phosphorylation. As shown in Figure 3E, Aβ1-42 treatment increased p-GSK3β (Y216) without changing the total GSK3β level. By contrast, Aβ1-40 treatment resulted in no change in either GSK3β or p-GSK3β.

Aβ1-42 treatment has also been reported to activate pro-caspase-3 [11] and enhance the cleavage of tau at Asp^421^, which results in accelerated aggregation. Consistent with these results, we also found increased Tau421 in Aβ1-42 treated cells. Western blots with an anti-active caspase-3 antibody revealed higher levels of active caspase-3 following Aβ1-42 treatment but not following Aβ1-40 treatment (Figure 3E and F).

### Aβ1-42, but not Aβ1-40, interferes with cytoskeleton formation in tau transgenic flies

**Figure 4:**
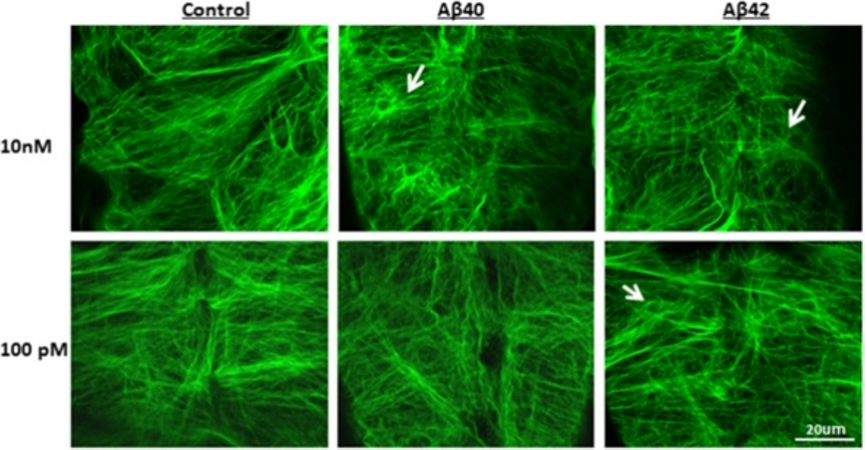
**Aβ** **1-42 disrupts cytoskeleton in transgenic fly at 100pM.** Aβ1-40 or Aβ1-42 was diluted in 4% BSA and injected into the body cavity of late embryo of GFP-tau (bovine Tau) transgenic fly (AEL-21-22 hr). 4 days later, the central nerve cord was dissected out, and mounted in PBS and imaged directly with a confocal microscope. Stacks of 20–40 0.5 μm confocal sections were generated. The results for each section were assembled as a stack. At 100pM, damage to the structure of cytoskeleton was observed in Aβ1-42 treated flies but not in flies receiving the Aβ1-40 treatment. At 10nM, both Aβ1-40 and Aβ1-42 led to disruption of the cytoskeleton.

Tau plays an important role in microtubule stabilization and organization. Changes in the cleavage, phosphorylation or aggregation of tau may directly lead to the disruption of microtubules architecture. To study the functional effects of Aβ on tau, we used a transgenic *Drosophila* model that expresses fluorescence GFP labeled tau (TauGFP) in subperineural glia cell. TauGFP labeled microtubule fibers are visible throughout the cytoplasm, emerging from the microtubule organizing center (MTOC) adjacent to the nucleus and extending to the cell cortex (Figure 4). Soluble Aβ1-40 or Aβ1-42 (100pM or 10nM) was microinjected into the body cavity of late embryo (AEL-21-22 hr). Four days later (3rd instar larvae), the central nerve cord was dissected out, mounted in PBS and imaged directly with a confocal microscope [12]. The microtubule cytoskeleton was easily visualized under confocal microscopy which allows fluorescence allowing us to measure changes of microtubule architecture as a result of the Aβ treatment. At 100pM, the microtubule organization of subperineural glia cell injected with Aβ1-42 was destabilized and clustered, while the microtubule architecture was normal in Aβ1-40 injected flies (Figure 4). When we increased the concentration of Aβ to 10nM, a compromised cytoskeleton was observed in both Aβ1-42 and Aβ1-40 injected flies.

### Aβ1-42 increases phosphorylated and insoluble tau, when compared with Aβ1-40, in the entorhinal cortex of P301S mice

To further examine the effect of Aβ on tau pathology in vivo, we performed intracerebral injections of Aβ into P301S mice (tau transgenic mouse model) at 8-months of age. At 8-months P301S mice have already developed tau pathology that increases as the animal age [13]. We targeted the entorhinal cortex as our injection site because neurons in the entorhinal cortex are more sensitive to Aβ-induced stress [14]. Unilateral injection of Aβ1-40 (2ug) into the left entorhinal cortex and Aβ1-42 (2ug) into the right entorhinal cortex allowed us to directly compare the effects of Aβ1–40 and Aβ1–42 in the same mouse.

**Figure 5:**
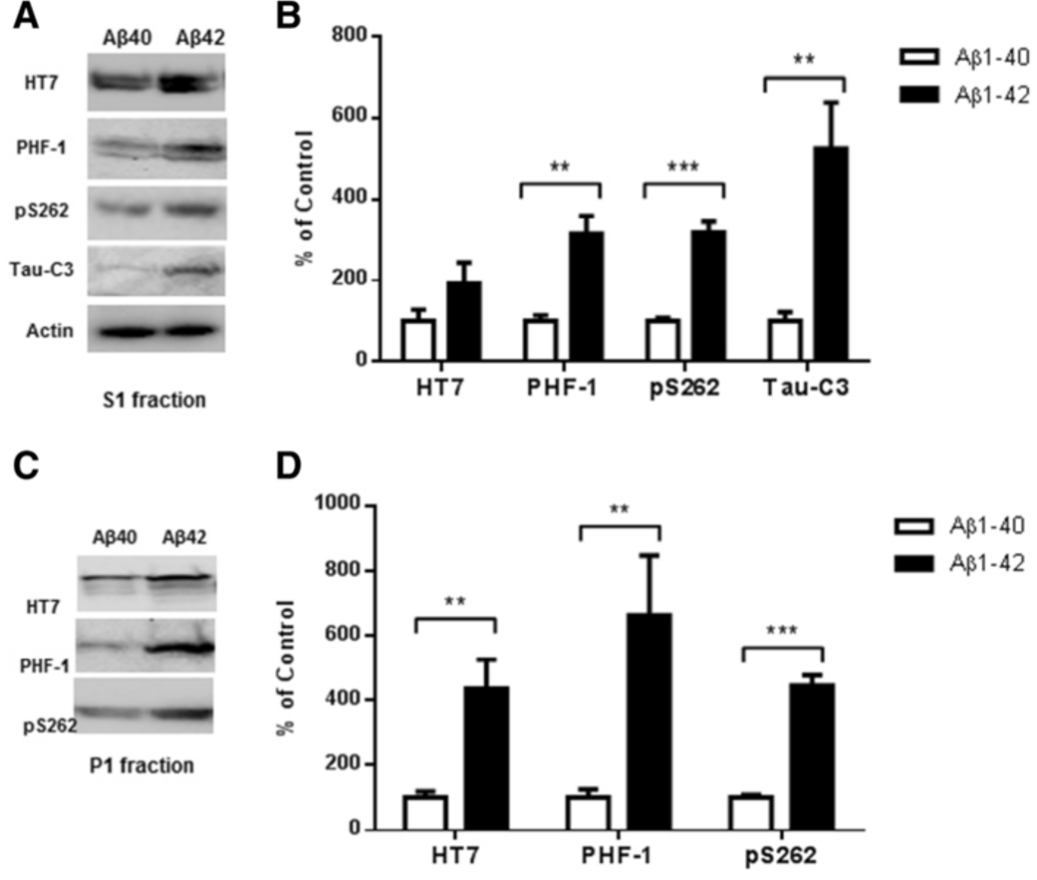
**Higher level of total tau, phospho-tau and cleaved tau in P301S mice receiving an Aβ** **1-42 injection into the entorhinal cortex as compared with Aβ** **1-40 injection.** 8 month old P301S mice were injected with Aβ1-40 into the left entorhinal cortex and Aβ1-42 into the right entorhinal cortex. 18 days after injection, the brain extracts were analyzed by western blot. **A)** Representative images of western blots of soluble tau (S1 fraction) detected with anti-tau antibodies and normalized to actin. **B)** Quantification of western blots in **(A)**. **C)** Representative images of western blots of insoluble tau (P1 fraction) detected with anti-tau antibodies. Both S1 brain fraction and P1 brain fraction show a significant increase in HT7, PHF-1 and pS262 signal in entorhinal cortex injected with Aβ1-42 compared with Aβ1-40 injected entorhinal cortex. Also more cleaved tau (Tau421) was detected in the S1 fraction from the Aβ1-42 injected brain. **D)** Quantification of western blots in **(C)**. Histographs show mean +/− standard error, n =3. *p <0.05; **p <0.01; ***p <0.005.

**Figure 6:**
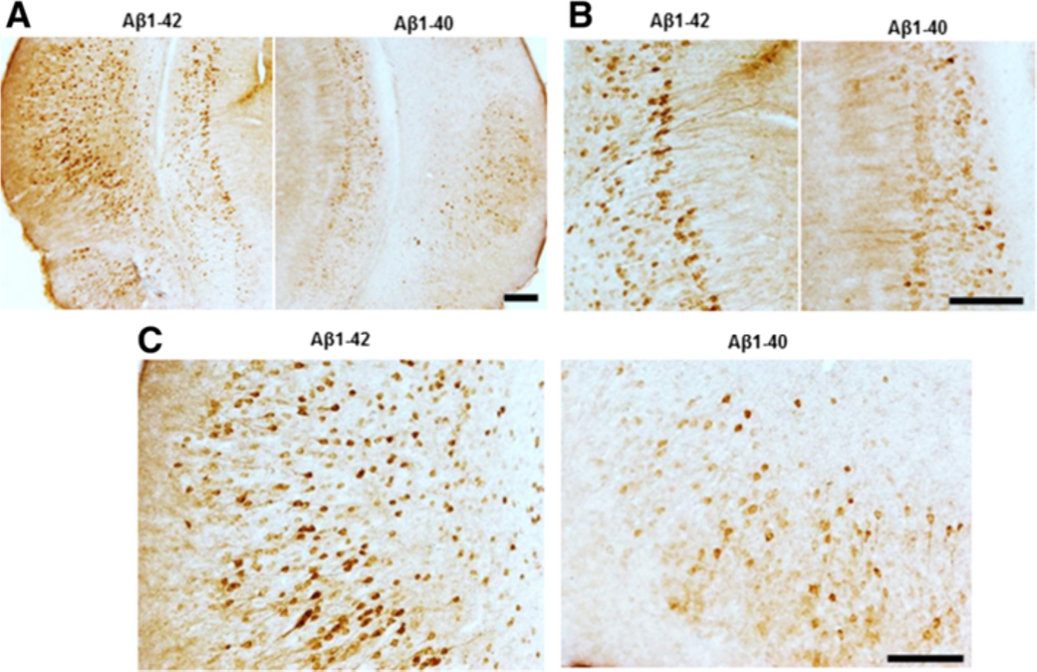
**Aβ** **1-42 treatment increases pathological tau recognized by AT180 when compared with Aβ** **1-40 treatment.** Brain sections were immunostained with AT180, which recognizes p-tau at Thr^231^. **(A)** Representative images for pathological tau detected with AT180. **(B)** Higher magnification showed significantly increased tau tangles in Aβ1-42 injected entorhinal cortex **(C)** and hippocampus **(B)**. Magnification: 4x for A; 10x for B and C. Scale bar: 100um.

Eighteen days after injection the mice were perfused with PBS and the brains were isolated, followed by fixation with 4% PFA. As shown in Figure 5A, significant increases in p-tau (recognized by PHF-1 and pS262) and Tau 421 (recognized by tau-C3) were observed in S1 fraction from Aβ1-42- injected entorhinal cortex compared to the Aβ1-40 injected entorhinal cortex. Western blot of P1 fraction from the brain extract (Figure 5C) showed that, a ≥4 fold increase in HT7, ≥6 fold increase in PHF1 and ≥4 fold increase in pS262 signal in Aβ1–42 treated as compared to Aβ1–40 treated entorhinal cortex. Immunohistochemistry with AT180, which recognizes phosphor-Thr^231^, suggests that injection of Aβ1-42 promotes greater phosphorylation and aggregation of tau when compared to Aβ1-40 in both the entorhinal cortex and hippocampus (Figure 6).

### Aβ1–42 promotes tau pathology while Aβ1–40 may inhibit tau pathology

**Figure 7:**
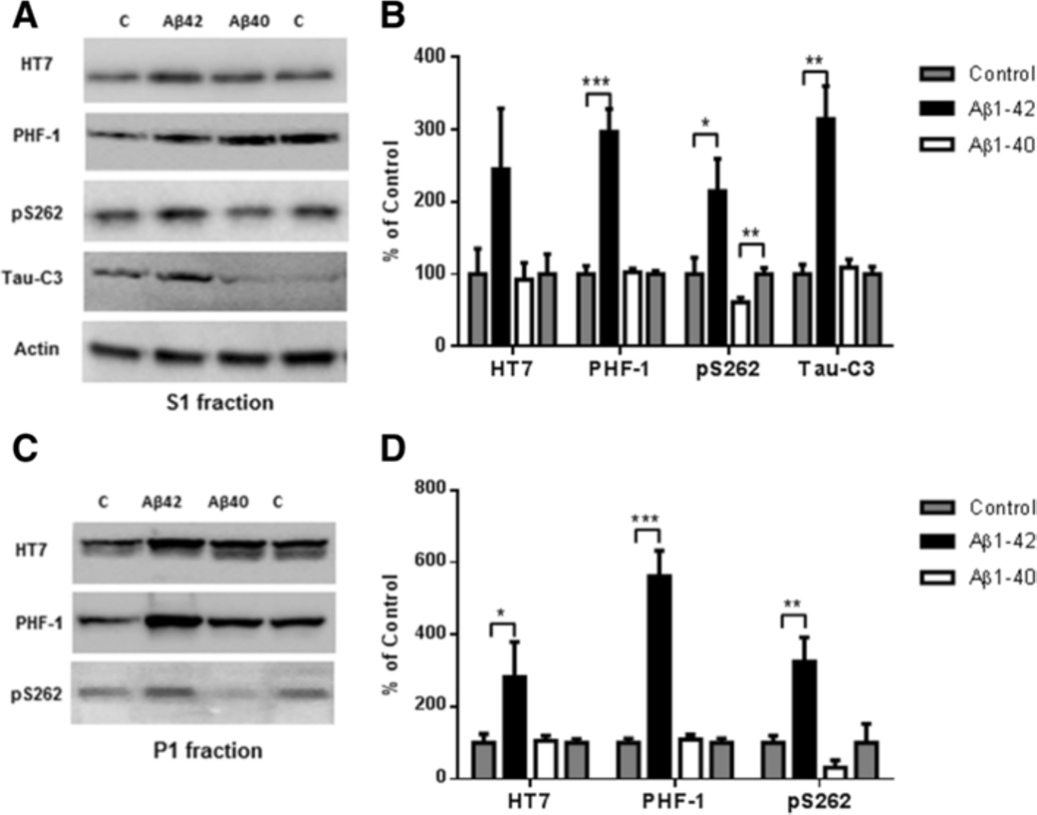
**Aβ** **1-42 promotes tau phosphorylation, cleavage and aggregation while Aβ** **1-40 does not.** 8 month old P301S mice (1N4R form with Prion promoter) were injected with saline into the left entorhinal cortex and Aβ1-42 (2ug) into the right entorhinal cortex or saline into the left entorhinal cortex and Aβ1-40 (2ug) into the right entorhinal cortex. 18 days after injection, the brain extracts were analyzed by western blot. **A)** Representative images of western blots of soluble tau (S1 fraction) detected with anti-tau antibodies and normalized to actin. **B)** Quantification of western blots in **(A)**. **C)** Representative images of western blots of insoluble tau (P1 fraction), detected with anti-tau antibodies. **D)** Quantification of western blots in **(C)** Aβ1-42 injection significantly induced tau phosphorylation (PHF-1 and pS262 epitope) in the S1 and P1 brain fractions. 3-fold increase in Tau421 was found in the soluble fraction **(A and B)**. Significant decrease in pS262 immunoreactivity was observed in S1 fraction from Aβ1-40 injected brain areas compared to control side. Histographs show mean +/− standard error, n =3. *p <0.05; **p <0.01; ***p <0.005.

The difference between Aβ1-40 and Aβ1-42-induced related tau pathology, shown in Figure 5, could be due to either an accelerating effect by Aβ1-42 or an inhibitory effect of Aβ1-40. To address this question, two groups of P301S mice were injected in the entorhinal cortex. One group was injected with PBS into the left entorhinal cortex and Aβ1-40 (2ug) into the right entorhinal cortex, a second group was injected with PBS into the left entorhinal cortex and Aβ1–42 (2ug) into the right entorhinal cortex. Eighteen days after injection, the mice were sacrificed and the whole hemibrains were dissected and analyzed by western blot. As shown in Figure 7A, there were higher levels of p-tau at Ser^262^, Ser^396^ and Ser^404^ in the Aβ1–42- injected side when compared to the contralateral PBS side (S1 fraction in Figure 7A and P1 fraction in Figure 7B). There were also higher levels of cleaved tau and insoluble tau after Aβ1-42 injection. We therefore conclude that Aβ1–42 accelerated tau pathology in P301S mice. For the Aβ1–40 treated group, we didn’t observe any differences in total tau, p-tau (PHF-1 specific) or cleaved tau. Interestingly, in the Aβ1-40-injected entorhinal cortex, we observed less phosphorylation at Ser^262^ in both S1 and P1 fraction.

## Discussion

Our data demonstrates that Aβ1-40 and Aβ1-42 have distinct effects on tau phosphorylation, aggregation and cleavage as shown in a variety of in vitro and in vivo models. Most cellular models previously used to study tau pathogenesis used a MTBR (microtubule binding domains) ta fragment instead of full-length tau. In recent years, increasing evidence has revealed that self-aggregation of tau into filaments is inhibited by the presence of intact N- and C-termini. The N-terminus is required for the binding of tau to the plasma membrane while cleavage at Asp421 (20 amino acids from C-terminus) significantly increases tau aggregation. Therefore, using full-length tau (2N4R, 441 amino acids) may be more relevant for studying the metabolism of tau, especially tau cleavage. The construct we used in our experiments, Tau441-YFP incorporates YFP to the C-terminus of tau which doesn’t interfere with the normal functions of the N-terminus of tau [15].

Tau is a highly soluble protein and is less prone to aggregate in many cell-based models [16]. To our knowledge, our study is the first to show that full-length tau can aggregate in certain cell type, SHSY5Y and C17.2 cells, as early as 24 h after transient transfection and without the addition of exogenous seeds. In addition, the rate of tau aggregation was not correlated with the level of tau expression because no aggregates formed in N2a cells at 24 h even though our N2a cells expressed a much higher level of full-length tau. For cells with lower degradation efficiency, tau aggregation may occur earlier, consistent with what we found in SHSY5Y and C17.2 cells. Even for cells with high degradation efficiency, continuous overexpression of tau may eventually overwhelm degradation ultimately leading to aggregation. Tau aggregates appeared by 72 h in the N2a cells and stained positively with Thioflavin-S, indicating that overexpression of tau is another factor that contributes to tau aggregation and subsequent fibrilization.

Among the many tau phosphorylation sites, Ser^262^ is the major site implicated in the abnormal functioning of tau in the AD brain [10, 16]. Our biochemical data, from treating stably transfected cell lines overexpressing Tau441-YFP with Aβ1-42, indicate increased phosphorylation at Ser^262^ in both Triton and SDS soluble fractions when compared to cells treated with Aβ1-40 and non-treated cells. Phosphorylation at Ser^262^ dramatically reduces the ability of tau to bind to microtubules, which ultimately results in microtubule destabilization. Microtubule destabilization affects synaptic vesicle transport and may disrupt synaptic function in vivo [17]. In transgenic Drosophila expressing both human Aβ1-42 and tau, Aβ1-42 specifically increases tau phosphorylation at Ser^262^ and enhances tau-induced neurodegeneration [18]. Co-expression of Aβ1-42 and tau carrying the non-phosphorylatable Ser262Ala mutation did not cause neurodegeneration, suggesting that the Ser^262^ phosphorylation is required for the pathogenic interaction between Aβ1-42 and tau [18]. We found that at low concentration, Aβ1-40 did not induce phosphorylation at Ser^262^ in either Triton or SDS soluble fractions in our cell model. This may explain why we do not see an increase in insoluble tau with Aβ1-40 treatment (Figure 2).

Aβ1-42 has been shown to induce tau phosphorylation and the formation of neurofibrillary tangles via a GSK-3β-dependent mechanism in both in vitro and in vivo studies [1, 19, 20]. Aβ42-induced neurotoxicity seems to depend on considerable levels of tau that can be inhibited by treatment with GSK-3β inhibitors [21]. The activity of GSK3β is also regulated positively by the phosphorylation of Tyr^216^. Up-regulation of GSK3β activity increases phosphorylation of tau at multiple sites and inhibition of GSK3β specifically decreases the phosphorylation of Ser^262^[22]. We found that Aβ1-42 activated GSK3β, but Aβ1-40 did not, which may explain the different effects of Aβ1-40 and Aβ1-42 on tau phosphorylation shown in Figure 3A and C. Our observation of increased Tau421 in Aβ1-42 treated cells, as well as previous studies on the correlation between caspase-3 activation and tau cleavage, indicate that the effects we observed following Aβ1-42 treatment on tau may be mediated by caspase-3 activation. In fact, western blot using a specific antibody to active caspase-3 indicated that Aβ1-42 activates pro-caspase-3 but no activation was observed with Aβ1-40 treatment. This may explain the marked difference we observed between the level of Tau421 in Aβ1-42 and Aβ1-40 treated cells (Figure 3).

In order to find out how Aβ directly affects the structure and function of tau in vivo, we used a tau-GFP transgenic Drosophila model. This is an ideal model because GFP-tagged tau can be used as a subcellular marker to study cytoskeletal dynamics in living cells. The latter avoids potential fixation artifacts and allows for the dynamic visualization of changes in the structure of the cytoskeleton. Another advantage of this model is that low concentrations of Aβ and short incubation times are sufficient to see the effects of Aβ on tau. Aβ1-42 injections at concentrations as low as 100pM caused cytoskeleton disruption in this model, but we did not see an effect on the cytoskeleton after exposure to low concentration of Aβ1-40. Interestingly, when we increased the concentration of Aβ to 10nM, we saw disrupted cytoskeletal structure in both Aβ1-40 and Aβ1-42-injected flies. Nonetheless, our data clearly show marked differential effects of Aβ1-42 and Aβ1-40 on tau function using live cell imaging in this tau transgenic fly model.

In vivo studies using a transgenic mouse model expressing P301S tau were carried out to further assess how the two main Aβ species may affect tau pathology in a vertebrate system. In AD, the formation of NFTs starts in the entorhinal cortex and spreads to the hippocampus and eventually to most cortical areas [23]. There are some advantages of injection of Aβ in the entorhinal cortex over other brain areas. First, unilateral injections can be carried out on both the ipsilateral and contralateral entorhinal cortex since there is no cross-talk between the left and right entorhinal cortex [24] which allows for a direct comparison of Aβ species in the same animal. Second, this approach allows us to study how Aβ and tau interact at the early stage of the disease. Third, expression of tau in entorhinal cortex can spread along anatomically connected networks to hippocampus [25]. Eighteen days after injection, biochemical analyses with soluble and insoluble fraction from injected brain tissues demonstrated that Aβ1–42 promotes tau cleavage, phosphorylation and aggregation. By contrast, treatment with Aβ1–40 showed no effect on the total tau or p-tau (recognized by PHF-1) level. Immunostaining with AT180 showed higher levels of p-tau (Thr^231^) in the entorhinal cortex (injection site), as well as in hippocampus, suggesting that pathological tau induced by Aβ1-42 in the entorhinal cortex may spread to other brain areas as reported by Gotz and coworkers [1]. The decrease observed in tau phosphorylation at Ser^262^ in the presence of Aβ1–40 suggests that Aβ1–40 may inhibit tau pathology in old tau transgenic mice that already have tangles. It will worth to try breed BRI-Aβ40 or BRI-Aβ42 mice with tau transgenic mice to see how Aβ1-40 or Aβ1- 42 affects tau pathogenesis in vivo. Also we are working on how combination of Aβ 1-40 and Aβ1-42 changes tau pathology compares to Aβ1-42 and study if Aβ1-40 inhibits tau pathology induced by Aβ1-42.

In conclusion, data from cell-based models, tau-GFP transgenic Drosophila and tau transgenic mouse models indicate that Aβ1–42 is clearly the pathogenic Aβ species with respect to inducing the “pathology”; i.e., increased cleavage, phosphorylation and aggregation of soluble wild-type tau. By contrast, Aβ1–40 has little to no effect on the pathogenesis. In fact, Aβ1–40 may even subserve a protective role in vivo as indicated by the decrease in phosphorylation at Ser^262^. Therapeutic strategies that preferentially target Aβ1–40 thereby increasing the ratio of Aβ1–42 to Aβ1–40 may actually be problematic. However, targeted Aβ treatments that lower the ratio of Aβ1–42 to Aβ1–40 may be of therapeutic benefit.

## Methods

### Aβ Preparation: Aβ_1–42_ and Aβ_1–40_

Dry peptide (1 mg) was pretreated with neat trifluoroacetic acid (1 mL), distilled under nitrogen, washed with 1,1,1,3,3,3-hexafluoro-2-propanol (1 mL), distilled under nitrogen, then dissolved in DMSO to 10 mM, and stored at −20°C [26].

### Cell culture and treatment

C17.2 cells (mouse neural progenitor cell line), a gift from Marc Diamond, were grown in DMEM, supplemented with 10% fetal bovine serum, 5% horse serum, and 1% pen/strep. SH-SY5Ycells (human neuroblastoma cell line) were cultured in DMEM/F12 medium supplemented with 10% FBS. N2a cells were grown in DMEM/OPTI-MEM supplemented with 5% FBS.

For transient transfections, cells were plated in 8-well Lab-Tek chamber slides (Nunc) and were transfected using Lipofectamine 2000 constructs (Invitrogen) according to the manufacturer’s recommendations.

For treatment: 200nM Aβ1–42 or Aβ1–40 was added to the medium and imaged at varied time points thereafter.

### Live cell imaging

A Live-cell imaging was performed on a Leica TCS SP5 spectral confocal microscope using an oil-immersion 63× lens. Observation of the cells with 514-nm laser was performed at low light/laser intensities to prevent photo-toxicity.

### Staining

For Thioflavin-S staining, cells were incubated with 0.025% Thioflavin-S (in 50% ethanol) for 5 min, rinsed with 50% ethanol and water, and cover slipped for imaging.

For insoluble tau: Cells fixed with 4% paraformaldehyde containing 1% Triton X-100 for 15 min to remove soluble proteins and fluorescence after extraction was recognized as insoluble tau. Fluorescence intensity was quantified with ImageJ.

### Sequential extraction and western blot

For generation of stable tau expressing cell lines, individual clones of N2a cells were selected in the presence of 500 μg/ml Geneticin after transfection, then picked and propagated in serum-DME supplemented with 200 μg/ml Geneticin.

N2a cells stably expressing tau-YFP were treated with 200nM Aβ1–42 or Aβ1–40. 24 h later, cells were collected and cell pellets were resuspended in 1% Triton lysis buffer and incubated on ice for 15 min. Following sonication, lysates were centrifuged at 12,000 × *rpm* for 20 min at 4°C. Supernatants were kept as “Triton fraction,” whereas pellets were resuspended, and sonicated in 1% SDS lysis buffer. After centrifugation at 12,000 × *rpm* for 20 min at room temperature, supernatants were saved as “SDS fraction.” Equal proportions of Triton and SDS fractions were resolved on 4–20% Tris-glycine midi gel, transferred to nitrocellulose membrane using the iBlot Dry Blotting System, and probed with specific antibodies in Table 1.

**Table 1:** **Antibodies**

Antibody	Epitope	Source
HT7	Tau (159-161aa)	Pierce (cat#MN1000)
pS262	p-Tau (phosphorylated at Ser262)	Invitrogen (cat# 44-750G)
AT180	p-Tau (phosphorylated at Ser231)	Pierce (cat#MN1040)
PHF-1	p-Tau (phosphorylated at Ser396 and 404)	Generous gift from Peter Davies
Tau-C3	Cleaved tau (cleaved at Asp421)	Millipore (cat#MAB5430)
Anti-caspase-3	Cleaved caspase-3	Cell signaling (cat#9662S)
Anti-GSK-3β	Total GSK3β	BD Sciences (610201)
Anti-GSK3β (phospho Y216)	p-GSK3β (phosphorylated at Tyr216)	Abcam (cat#ab75745)

### Tau-transgenic fly

UAS-tau-GFP transgenic fly line (tau-GFP is driven by SPG-specific moody-Gal4 driver) was used for injection and live imaging. 100 pm or 10nM Aβ1–42 or Aβ1–40 was injected into the body cavity of late embryo (AEL-21-22 hr), and they were allowed to developed until 3rd instar larvae (about 4 days). Then the central nerve cord was dissected out, and mounted in PBS and imaged directly. All confocal images were acquired using a Zeiss LSM 710 system. Stacks of 20–40 0.5 μm confocal sections were generated. The results for each section were assembled as a stack.

### Stereotaxic injections of Aβ into entorhinal cortex

P301S mice were purchased from Jackson Labs. All experiments were approved by the Institutional Animal Care and Use Committee at Weill-Cornell Medical College. P301S mice (Jackson labs) were anesthetized with isofluorane and placed in a Stoelting stereotaxic instrument (Stoelting Co., Wood Dale, Illinois) and an incision was made along the midline. The coordinates for injection of Aβ42 were determined with reference to the bregma at position: AP, −3.6 mm; L, ±3.8 mm; DV, −4.5 mm. Using a 5 μl Hamilton syringe (Hamilton Inc., Bonaduz, Switzerland) driven by a mini pump (Motorized Stereotaxic Injector, Stoelting), a total volume of 2 μl of the Aβ1-42, Aβ1-40 or Saline was injected with an injection speed of 0.25 μl/min unilaterally into the entorhinal cortex. The needle was kept in the injection site for 5 min and then slowly withdrawn to prevent a backflow of the Aβ preparation.

### Biochemical analysis for brain extract

Brain tissues were weighed and homogenized in 10 volumes of homogenization buffer (Tris-buffered saline (TBS), pH 7.4, containing 1× protease and phosphatase inhibitor mixture with 2 mM EGTA). The homogenized samples were spun at 21,000 × *g* for 20 min, the supernatants were centrifuged at 100,000 × *g* for 1 h at 4°C to obtain insoluble pellet and supernatant (S1 fraction). The insoluble pellet (P1 fraction) was resuspended in 1% sarkosyl in H buffer (10 mM Tris-HCl, 1 mM EGTA, 0.8 M NaCl, 10% sucrose, and protease inhibitor mixture, pH 7.4) and sonicated. The solution was clear and label as P1, diluted in H2O for ELISA [27]. S1 or P1 fraction was separated on 4–20% Tris-glycine midi gel, transferred to iblot gel nitrocellulose using the iblot Dry Blotting System, and probed with HT7, PHF-1, pS262 and tau-C3.

### Immunohistochemistry

Brains were placed in 10% buffered formalin, followed by 30% sucrose. These sections were subsequently stained using phosphor-tau antibody- AT180. Secondary antibody was applied, and slides were then incubated with avidin-biotin complex reagent for 5 min. After rinsing, slides were treated with the chromogen 3,3′-diaminobenzidine (Vector Laboratories, SK-4100) to allow visualization.

## Abbreviations

AD: Alzheimer’s disease
Aβ: Amyloid beta
NFT: Neurofibrillary tangles.
